# Clinical and Pathophysiological Characteristics of Cirrhotic Patients with Grade 1 and Minimal Hepatic Encephalopathy

**DOI:** 10.1371/journal.pone.0146076

**Published:** 2016-01-08

**Authors:** Karen Louise Thomsen, Jane Macnaughtan, Giovanni Tritto, Rajeshwar P. Mookerjee, Rajiv Jalan

**Affiliations:** Liver Failure Group, UCL Institute for Liver and Digestive Health, University College London Medical School, London, United Kingdom; University of Navarra School of Medicine and Center for Applied Medical Research (CIMA), SPAIN

## Abstract

**Background and Aims:**

EASL/AASLD hepatic encephalopathy (HE) guidelines proposed the alternative use of the term ‘Covert HE’ combining minimal HE (mHE) and Grade 1 HE into a single entity. However, longitudinal data to indicate that these are indeed a single entity are lacking. The aims of this study were to determine whether the occurrence of complications of cirrhosis requiring hospital admission and mortality were similar in these sub-groups of patients.

**Methods:**

Clinically-stable cirrhotic patients (n = 106) with no previous history of ‘Overt HE’ were included over a 2-year period and classified as having no HE (n = 23), mHE (n = 39) or Grade 1 HE (n = 44). Standard biochemistry, venous ammonia, bacterial DNA and neutrophil function were measured at inclusion and the patients were followed for a mean of 230±95 days.

**Results:**

Patients with Grade 1 HE had significantly more complications requiring hospitalisation (infection 9/18/34%; HE 4/8/18%; other 13/10/11%; P = 0.02) and significantly greater mortality (4/5/20%; P = 0.04) compared to patients with no HE or mHE respectively. Patients with mHE and grade 1 HE had similar ammonia levels, but higher than the no HE group (P<0.001). MELD score was similar between groups but Grade 1 HE patients had increased frequency of bacterial translocation (P = 0.06) and neutrophil spontaneous respiratory burst (P = 0.02) compared to patients with mHE.

**Conclusions:**

The results of this study show for the first time that ‘Covert HE’ is a heterogeneous entity with significantly greater hospitalisations and mortality in the Grade 1 HE patients compared with mHE. Further prospective longer-term studies are required before EASL/AASLD guidance is fully implemented.

## Introduction

The presence of hepatic encephalopathy (HE) in patients with cirrhosis increases mortality with the worsening of HE grade [1–4]. Previous studies have been limited to either patients with ‘overt HE’ comparing grade 1–2 HE and grade 3–4 HE or to studies in patients with minimal hepatic encephalopathy (mHE) only. mHE patients are at increased risk of developing ‘overt HE’ compared to cirrhotic patients with no HE [5, 6] and have been shown to have a poor quality of life [7]. Bajaj et al and the recent EASL/AASLD guidelines on HE proposed the alternative use of the term ‘Covert HE’ combining mHE and Grade 1 HE into a single entity [8, 9]. The rationale for this proposal was that it is often difficult to diagnose Grade 1 HE because the diagnostic criteria are difficult to apply in clinical practice. The HE guidelines recommended a data-driven approach to evaluate whether the term ‘Covert HE’ is informative and clinically valuable as longitudinal data to indicate whether these are indeed a single entity are lacking.

Intercurrent complications of cirrhosis such as ascites, variceal bleeding, infection and HE are predictors of poor prognosis in cirrhotic patients. Infection is a common reason for hospital admission and progression to HE and death in cirrhotic patients. Impaired neutrophil function is observed in these patients [10] and ammonia has been shown to play a part by inducing impaired phagocytosis and increased neutrophil spontaneous oxidative burst [11]. The grade of HE is proposed to be associated with ammonia levels [12], although a consistent correlation between plasma ammonia levels and the grade of HE is not observed; a synergetic effect of inflammation and infection is also of importance [13, 14].

The aims of this prospective study were to determine whether the two entities, mHE and grade 1 HE are clinically and pathophysiologically homogeneous. We hypothesised that patients with grade 1 HE had more liver related complications requiring hospitalisation compared to patients with mHE. The occurrence of complications and mortality were considered as the main end-points of the study. For mechanistic linkage, ammonia levels, inflammatory markers and neutrophil function were also evaluated and compared between the two sub-groups of cirrhotic patients.

## Patients and Methods

This study was performed between 2008 and 2010 at University College London Hospitals. Ethics approval was obtained from University College London Hospitals Ethics Committee and all the participants provided written informed consent in accordance with the Helsinki Declaration. The study was as a part of a protocol set up to define the role of neutrophil dysfunction in cirrhosis and data from 8 of the patients included here have been published previously [15]. None of the HE data described here have been published previously.

### Subjects, study design and ethics

Patients were included if they were >18 and <75 years of age and had a clinical, radiological, or histological diagnosis of cirrhosis. Exclusion criteria were severe complications of liver disease including gastrointestinal bleeding, hepatorenal syndrome, hepatic encephalopathy (grade 2 or higher), bacterial infection, spontaneous bacterial peritonitis, treatment with systemic antibiotics, probiotics or norfloxacin for selective gut decontamination, hepatic or extrahepatic malignancy, any immunomodulatory or steroid therapy, any organ failure within the past 30-days. Patients known to be drinking alcohol at the time of study inclusion were also excluded. Liver function tests, white blood cell count (WBC), venous ammonia, bacterial DNA, neutrophil respiratory burst and phagocytosis were measured at inclusion and the patients were followed up to a year for the occurrence of complications requiring hospitalisation and death.

### Diagnosis of Grade 1 HE and mHE

The presence or the absence of Grade 1 HE was established by means of careful neurological examination using the West-Haven criteria. Each patient was examined by one hepatologist (RJ). The diagnosis was based on the neurological findings of lack of awareness, euphoria or anxiety, shortened attention span and/or impaired performance of addition. EEG, psychometric hepatic encephalopathy score (PHES) and critical flicker frequency (CFF) were used in screening for mHE. Patients were classified as having mHE if at least one of the three tests was abnormal. EEG was recorded for 10-minutes, in a relaxed condition with eyes-closed and using the standard 21 electrodes. EEGs were classified as normal or abnormal based on the spectral criteria described previously [16]. Psychometric performance was measured using the PHES test (number connection tests A and B, the digit symbol subtest of the Wechsler adult intelligence scale, line tracing and serial dotting tests) as described previously [17]. The tests were scored using age and education-adjusted UK scores. The PHES test were described as abnormal if the score was <-4 [17]. CFF was measured using the ‘Hepatonorm analyzer’ and abnormality was defined using a cut-off of < 39 [18].

### Biochemical analyses

Blood samples for bilirubin, albumin, INR, sodium, creatinine, WBC, and ammonium were analyzed immediately following collection by routine analytical methods. Blood samples for the assessment of bacterial DNA, neutrophil respiratory burst and phagocytosis were aseptically collected into pyrogen-free tubes, placed immediately on ice, centrifuged, separated and stored at -80°C until analysis.

### Bacterial DNA

Bacterial DNA was assessed using specific polymerase chain reaction for the conserved region of the 16S ribosomal RNA prokaryote gene as previously described [19].

### Neutrophil isolation

Neutrophils were harvested from healthy volunteers. Patient plasma was incubated with these cells as previously described [10]. The Phagoburst kit (Orpegen Pharma, Heidelberg, Germany) was used to determine the percentage of neutrophils that produce reactive oxidants and the Phagotest (Orpegen Pharma) was used to measure phagocytosis by using FITC-labelled opsonized *E*. *coli* bacteria as previously described (FACS Canto II, BD Bioscience) [10].

### Statistics analysis

Variables were tested for a normal distribution using qq-plots and histograms. Variables showing skewed distributions were logarithmically transformed for further analysis. Differences across categorical variables were accessed using Pearson χ^2^ test, whereas continuous variables were tested using the One Way Analysis of Variance on Ranks adjusted with a Bonferroni correction for multiple testing. Differences between episodes of complications or death in each group were tested using the log-rank test. Univariate analysis was carried out to identify the baseline factors associated with occurrence of complications or death. A logistic regression model was then fitted including all the significant potential predictors from the univariate analysis. Categorical variables are presented as frequencies and percentages, continuous parameters as mean ± SD. P-values <0.05 were considered statistically significant.

## Results

### Patient characteristics

One-hundred-and-six outpatients with clinically stable liver cirrhosis (age 58±10 years; 67 men (63%); Child-Pugh class A: 5%, B: 67%, C: 28%; MELD score 15±6) with no previous history of overt HE were prospectively included over a 2-year period. The aetiologies included viral (n = 44), alcohol (n = 43), mixed viral plus alcohol (n = 13), primary biliary cirrhosis (n = 2), non-alcohol steatohepatitis (n = 1), or unknown (n = 3). The severity of liver disease was assessed by the Model for End-Stage Liver Disease (MELD) [20] and the Child-Pugh (C-P) score [21]. Forty-four patients were diagnosed as having Grade 1 HE based on the West Haven criteria. Of the 44 grade 1 HE patients, 14 (32%) showed lack of awareness, 18 (41%) experienced euphoria or anxiety, 22 (50%) had shortened attention span and 19 (43%) showed impaired performance of addition. 22 (50%) of the patients had one symptom, 17 (38%) had two symptoms, 3 (7%) had three symptoms and 2 (5%) had all four symptoms. None of the patients classified as ‘unimpaired’ or ‘mHE’ showed these signs or symptoms. Thirty-nine patients were classified as having mHE based on at least one abnormal test (EEG, PHES, CFF). Of the 39 mHE patients, 19 (49%) had one abnormal test, 13 (33%) had two abnormal tests and 7 (18%) had abnormal outcomes in all three tests. Twenty-three patients were classified as being unimpaired. The patients in the 3-cohorts were followed up for a mean of 234±99 (no HE), 231±100 (mHE) and 227±89 (grade 1 HE) days.

The MELD score was similar in the three groups, whereas mHE patients had higher C-P scores compared to Grade 1 HE patients (P = 0.005). Also, there was a significant difference in extent of ascites; with 98% of Grade 1 HE patients having moderate-severe ascites (P = 0.003) (Table 1). No differences between the three groups in bilirubin, INR, albumin, sodium, creatinine or WBC count levels were observed. Ammonia levels were similar in patients with mHE and Grade 1 HE but significantly lower in the no HE group (P<0.001, respectively) (Table 1*)*. Ammonia levels were higher in patients with abnormal EEG compared to patients with abnormal PHES test (P<0.001) (Table 2).

**Table 1:** Baseline characteristics of patients with cirrhosis according to hepatic encephalopathy classification.

	Unimpaired	mHE	Grade 1 HE	P-value	P-value
	n = 23	n = 39	n = 44	(all 3 groups)	(mHE/grade 1)
Sex: m/f (%)	74/26	62/38	59/41	P = 0.47	
Age (year)	59 ± 6	58 ± 10	58 ± 12	P = 0.79	
MELD	15 ± 6	14 ± 6	16 ± 6	P = 0.12	
C-P score: A/B/C (%)	0/70/30	8/49/43	4/82/14	P = 0.019	P = 0.005
Ascites: 0/1/2 (%)	17/17/65	18/15/67	0/2/98	P = 0.003	P = 0.001
Abnormal EEG n (%)	0	21 (54)	24 (55)	P<0.001	P = 0.95
Abnormal PHES n (%)	0	24 (62)	40 (93)	P<0.001	P = 0.001
Abnormal CFF n (%)	0	21 (68)	36 (84)	P<0.001	P = 0.19
Bilirubin (μmol/L)	76 ± 97	85 ± 88	65 ± 49	P = 0.50	
INR	1.5 ± 0.3	1.5 ± 0.4	1.5 ± 0.3	P = 0.98	
Albumin (g/dL)	2.8 ± 0.7	2.9 ± 0.5	2.9 ± 0.5	P = 0.67	
Ammonia (μmol/L)	48 ± 11	61 ± 14	62 ± 12	P<0.001	P = 0.53
Sodium (mmol/L)	136 ± 5	136 ± 6	135 ± 5	P = 0.58	
Creatinine (μmol/L)	91 ± 60	69 ± 39	81 ± 43	P = 0.18	
WBC count (x 10^9^/L)	5.0 ± 2.2	6.5 ± 3.1	7.4 ± 4.8	P = 0.05	
Bacterial DNA n (%)	5 (22)	14 (36)	25 (57)	P = 0.01	P = 0.06
Neut. resp. burst (%)	13 ± 11	13 ± 14	22 ± 22	P = 0.03	P = 0.02
Phagocytosis (GMFI)	84 ± 15	81 ± 13	78 ± 10	P = 0.16	
Complications n (%)	6 (26)	14 (36)	28 (64)	P = 0.02	P = 0.01
− Infections n (%)	2 (9)	7 (18)	15 (34)	P = 0.04	P = 0.10
− HE n (%)	1 (4)	3 (8)	8 (18)	P = 0.16	
− Variceal bleed n (%)	1 (4)	2 (5)	2 (5)	P = 0.99	
− Ascites n (%)	1 (4)	2 (5)	3 (7)	P = 0.90	
− Unknown n (%)	1 (4)	0 (0)	0 (0)	P = 0.16	
Death n (%)	1 (4)	2 (5)	9 (20)	P = 0.04	P = 0.04

HE = hepatic encephalopathy, MELD = Model for End-Stage Liver Disease, C-P score = Child-Pugh score, PHES = psychometric hepatic encephalopathy score, CFF = critical flicker frequency, WBC = white blood cell count, GMFI = geometric mean of fluorescence intensity.

**Table 2:** Baseline characteristics of patients with cirrhosis according to test abnormalities.

		Abnormal	Abnormal	2–3 abnormal tests	P-value
	Unimpaired	EEG only	PHES only	(incl. PHES)*	(all groups)
	n = 23	n = 10	n = 11	n = 53	
Sex: m/f (%)	74/26	40/60	64/36	62/38	P = 0.33
Age (year)	59 ± 6	63 ± 8	51 ± 7	59 ± 12	P = 0.04
MELD	15 ± 6	15 ± 5	14 ± 4	16 ± 7	P = 0.80
Ascites: 0/1/2 (%)	17/17/65	10/20/70	9/27/64	7/4/89	P = 0.14
Grade 1 HE n (%)	0 (0)	1 (10)	4 (36)	36 (68)	P<0.001
Bilirubin (μmol/L)	76 ± 97	55 ± 28	55 ± 30	80 ± 83	P = 0.67
INR	1.5 ± 0.3	1.5 ± 0.4	1.4 ± 0.2	1.5 ± 0.3	P = 0.91
Albumin (g/dL)	2.8 ± 0.7	3.0 ± 0.3	3.1 ± 0.5	2.9 ± 0.5	P = 0.62
Ammonia (μmol/L)	48 ± 11	71 ± 12	53 ± 6	61 ± 14	P<0.001
Sodium (mmol/L)	136 ± 5	136 ± 7	136 ± 5	135 ± 5	P = 0.84
Creatinine (μmol/L)	91 ± 60	82 ± 36	69 ± 19	78 ± 48	P = 0.58
WBC count (x 10^9^/L)	5.0 ± 2.2	5.6 ± 3.1	4.8 ± 2.5	7.6 ± 4.4	P = 0.01
Bacterial DNA n (%)	5 (22)	4 (40)	7 (63)	23 (43)	P = 0.11
Neut. resp. burst (%)	13 ± 11	19 ± 18	26 ± 22	17 ± 19	P = 0.21
Phagocytosis (GMFI)	84 ± 15	81 ± 10	80 ± 14	79 ± 11	P = 0.41
Complications n (%)	6 (26)	3 (30)	3 (27)	32 (60)	P = 0.02
− Infections n (%)	2 (9)	1 (10)	0 (0)	16 (30)	P = 0.09
− HE n (%)	1 (4)	0 (0)	2 (18)	9 (17)	P = 0.25
− Others n (%)	3 (13)	2 (20)	1 (9)	7 (13)	P = 0.81
Death n (%)	1 (4)	0 (0)	0 (0)	11 (21)	P = 0.05

*current recommendation for diagnosis of mHE.

Left out ‘abnormal critical flicker frequency (CFF) only’ because of only 3 patients in the group.

PHES = psychometric hepatic encephalopathy score, MELD = Model for End-Stage Liver Disease, HE = hepatic encephalopathy, WBC = white blood cell count, GMFI = geometric mean of fluorescence intensity.

### Complications and mortality

**Fig 1:**
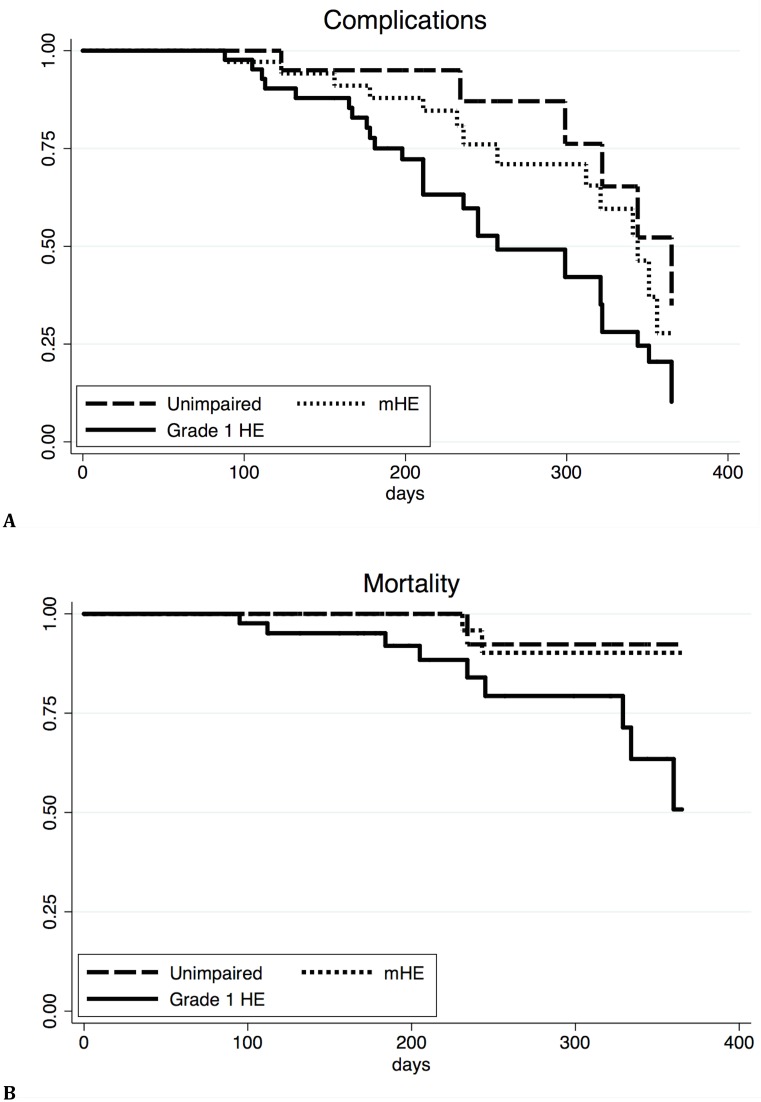
Time from inclusion to development of first liver related complication requiring hospitalisation (A) and survival curves for patients with no HE, mHE and Grade 1 HE (B). Dashed line depicts unimpaired patients (n = 23), dotted line patients with mHE (n = 39) and solid line patients with Grade 1 HE (n = 44) (P = 0.02 (A) and P = 0.04 (B)).

The three groups had significantly different rates of complications requiring hospital admission (unimpaired/ mHE/ Grade 1 HE: 26/ 36/ 64% respectively; P = 0.02) (Fig 1A). Patients with Grade 1 HE had more complications compared to patients with no HE and mHE (P = 0.01). Infection was the most common complication requiring hospitalisation (unimpaired/ mHE/ Grade 1 HE: 9/ 18/ 34% respectively; P = 0.04), followed by HE (unimpaired/ mHE/ Grade 1 HE: 4/ 8/ 18% respectively; NS), and others (variceal bleeding, ascites) (unimpaired/ mHE/ Grade 1 HE: 13/ 10/ 11%; respectively; NS). MELD score, HE grade and bilirubin level, ammonia and neutrophil respiratory burst were significantly higher in patients developing complications requiring hospitalisation than in those who did not, at univariate analysis (Table 3). These variables were introduced into a logistic regression model disclosing HE grade 1 (OR = 3.8; 95%CI: 1.05–14.04; P = 0.04) and bilirubin (OR = 1.01; 95%CI: 1.00–1.02; P = 0.01) as independent predictors of developing complications requiring hospitalisation.

**Table 3:** Baseline characteristics of patients with cirrhosis developing complications requiring hospitalisation (Yes) and those who did not (No) and of survivors versus non-survivors.

	Complications			Death		
	Yes (n = 48)	No (n = 58)	P-value	Yes (n = 12)	No (n = 94)	P-value
Sex: m/f (%)	60/40	66/34	P = 0.59	75/25	62/38	P = 0.37
Age (year)	57 ± 11	59 ± 10	P = 0.24	57 ± 10	58 ± 10	P = 0.80
MELD	17 ± 6	14 ± 5	P = 0.005	16 ± 5	15 ± 6	P = 0.77
Ascites: 0/1/2 (%)	8/4/88	12/16/72	P = 0.11	8/0/92	11/12/77	P = 0.42
No/mHE/grade 1 HE (%)	13/29/58	29/43/28	P = 0.005	8/17/75	24/39/37	P = 0.04
Bilirubin (μmol/L)	95 ± 97	58 ± 50	P = 0.01	91 ± 91	73 ± 75	P = 0.42
INR	1.6 ± 0.3	1.4 ±0.3	P = 0.05	1.6 ± 0.4	1.5 ± 0.3	P = 0.09
Albumin (g/dL)	2.9 ± 0.5	2.9 ± 0.6	0 = 0.59	2.8 ± 0.6	2.9 ± 0.5	P = 0.49
Ammonia (μmol/L)	62 ± 14	56 ± 13	P = 0.03	67 ± 18	57 ± 13	P = 0.03
Sodium (mmol/L)	135 ± 5	135 ± 5	P = 0.95	134 ± 5	135 ± 5	P = 0.24
Creatinine (μmol/L)	80 ± 44	78 ± 47	P = 0.83	58 ± 21	81 ± 48	P = 0.10
WBC count (x 10^9^/L)	6.9 ± 3.6	6.2 ± 4.0	P = 0.31	7.0 ± 2.0	6.5 ± 4.0	P = 0.67
Bacterial DNA n (%)	22 (46)	22 (38)	P = 0.41	5 (42)	39 (41)	P = 0.99
Neut. resp. burst (%)	22 ± 19	13 ± 15	P = 0.005	33 ± 22	15 ± 16	P<0.001
Phagocytosis (GMFI)	78 ± 12	82 ± 13	P = 0.13	75 ± 11	81 ± 13	P = 0.14

MELD = Model for End-Stage Liver Disease, HE = hepatic encephalopathy, WBC = white blood cell count, GMFI = geometric mean of fluorescence intensity.

Mortality was significantly different between the three groups (unimpaired/ mHE/ Grade 1 HE: 4/ 5/ 20% respectively; P = 0.04) with Grade 1 HE patients having greater mortality than mHE patients (P = 0.04) (Fig 1B). Univariate analysis demonstrated significantly higher baseline levels of HE grade, ammonia and neutrophil respiratory burst in patients who died compared to those who survived, whereas bacterial DNA positivity was not significantly associated with mortality (Table 3). However, only neutrophil respiratory burst (OR = 1.03; 95%CI: 1.00–1.07; P<0.05) was an independent predictor of mortality in a logistic regression model.

### Bacterial DNA, neutrophil respiratory burst and phagocytosis

A difference in the prevalence of bacterial DNA positivity between the three groups was observed (P = 0.01) (Table 1). Patients with Grade 1 HE had increased rates of detectable bacterial DNA (57%) compared to patients with no HE (22%; P = 0.02) and mHE (36%; P = 0.06). However, the presence or the absence of bacterial DNA did not predict which patients were likely to develop complications or die (Table 3).

**Fig 2:**
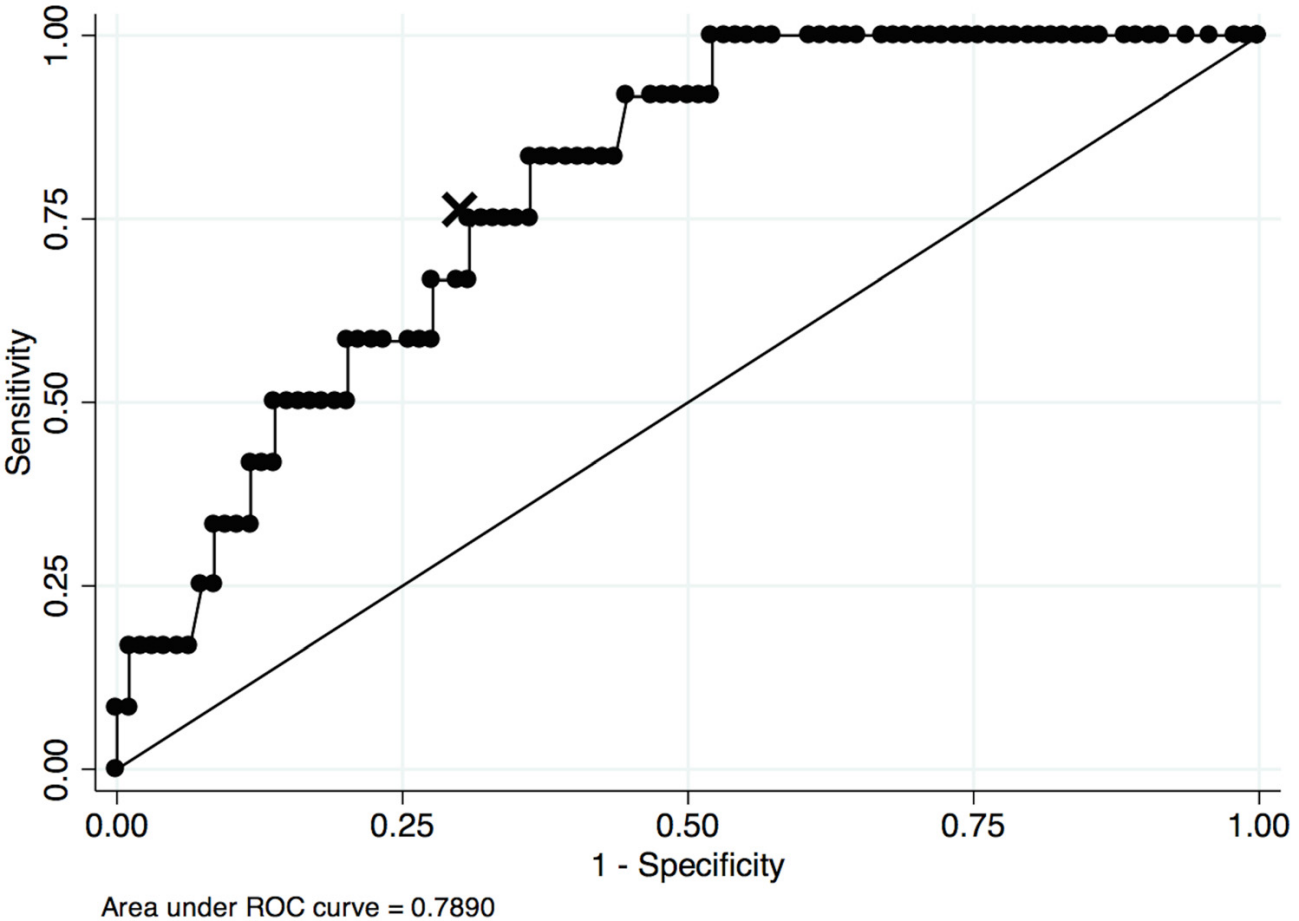
Receiver operating characteristic (ROC) of analyses of neutrophil respiratory burst. Receiver operating characteristic (ROC) of analyses of neutrophil respiratory burst as a predictor for mortality in cirrhotic patients. Selected cut-off for neutrophil respiratory burst 19.8% marked with X.

**Fig 3:**
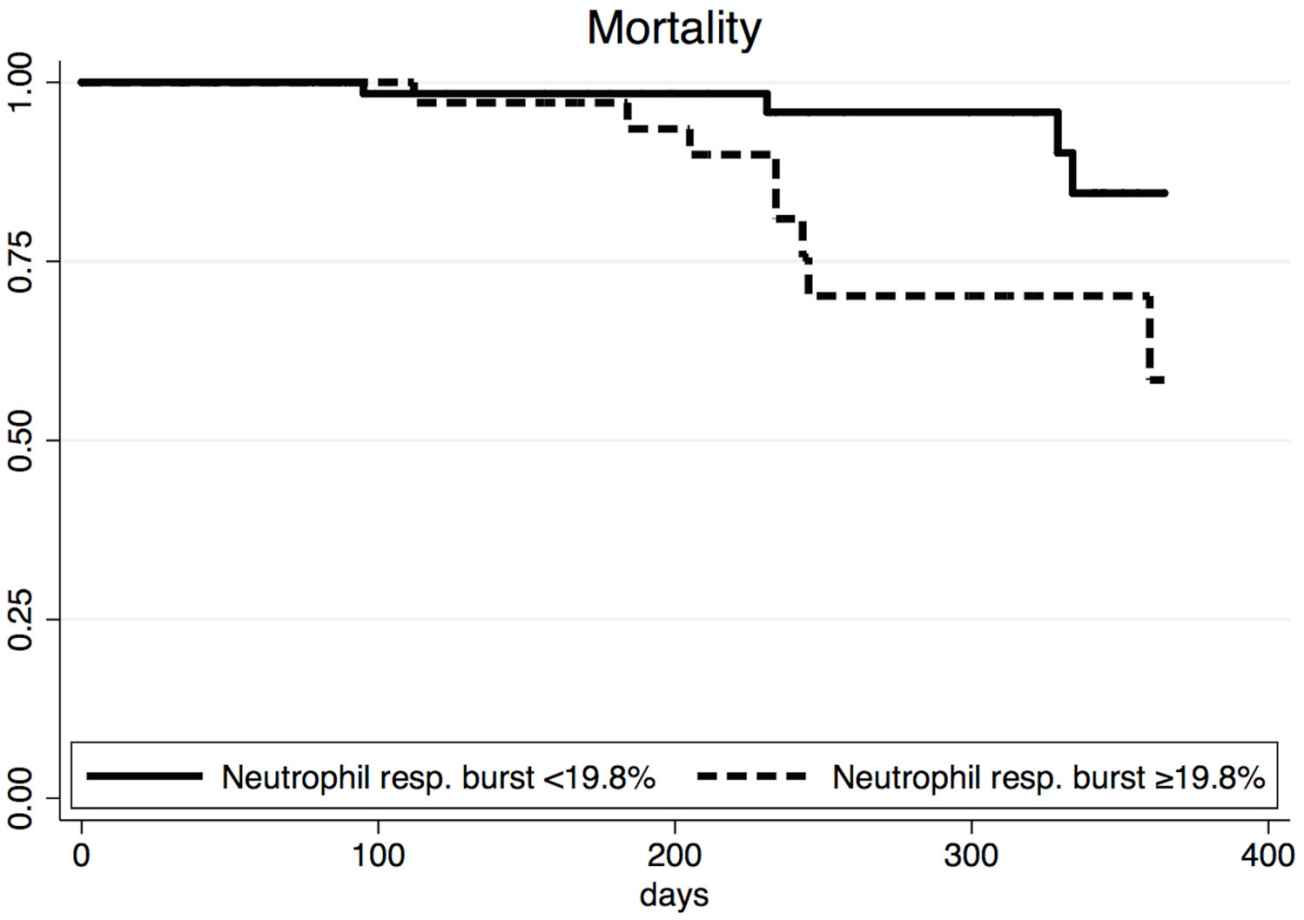
Survival curves for patients according to neutrophil respiratory burst. Survival curves for patients with neutrophil respiratory burst <19.8% (n = 69, solid line) and ≥19.8% (n = 37, dashed line) (P = 0.03).

There was no difference in neutrophil phagocytosis between the three groups, whilst spontaneous neutrophil respiratory burst was higher in the patients with Grade 1 HE (22±22%) compared to patients with no HE (13±11%) and mHE (13±14%) (P = 0.03) (Table 1). Higher respiratory burst was associated with an increased risk of cirrhosis complications requiring hospital admission and with mortality (Table 3). ROC curve analysis showed that spontaneous respiratory burst was indeed a good predictor of mortality (AUROC 0.79) (Fig 2). A cut off of 19.8% had sensitivity of 75% and specificity of 69%. Mortality was significantly higher in the group of patients with values ≥19.8% compared to the group with values <19.8% (22 vs. 6%; P = 0.03) (Table 4 and Fig 3).

**Table 4:** Baseline characteristics of patients with cirrhosis according to neutrophil respiratory burst.

Neutrophil	< 19.8%	≥ 19.8%	P-value
respiratory burst	(n = 69)	(n = 37)	
Sex: m/f (%)	65/35	59/41	P = 0.56
Age (year)	59 ± 10	57 ± 10	P = 0.28
MELD	15 ± 6	16 ± 6	P = 0.37
Ascites: 0/1/2 (%)	13/10/77	5/11/84	P = 0.47
No/mHE/grade 1 HE (%)	23/42/35	19/27/54	P = 0.15
Bilirubin (μmol/L)	73 ± 71	78 ± 87	P = 0.73
INR	1.5 ± 0.4	1.5 ± 0.3	P = 0.79
Albumin (g/dL)	2.9 ± 0.6	2.9 ± 0.5	P = 0.44
Ammonia (μmol/L)	58 ± 13	60 ± 16	P = 0.42
Sodium (mmol/L)	135 ± 5	135 ± 5	P = 0.77
Creatinine (μmol/L)	78 ± 49	80 ± 40	P = 0.81
WBC count (x 10^9^/L)	6.2 ± 3.7	7.2 ± 4.0	P = 0.19
Bacterial DNA n (%)	28 (41)	16 (43)	P = 0.79
Phagocytosis (GMFI)	82 ± 13	78 ± 12	P = 0.14
Complications n (%)	24 (35)	24 (65)	P = 0.03
Mortality n (%)	4 (6)	8 (22)	P = 0.03

MELD = Model for End-Stage Liver Disease, HE = hepatic encephalopathy, WBC = white blood cell count, GMFI = geometric mean of fluorescence intensity.

## Discussion

The results of this study show that patients with Grade 1 HE have significantly more cirrhosis-related complications and higher mortality compared to patients with mHE indicating that ‘Covert HE’ is indeed a heterogeneous entity comprising clinically and pathophysiologically distinct syndromes. In fact, the occurrence of Grade 1 HE was a strong predictor of significantly worsened prognosis, whilst the clinical outcomes of patients with mHE were similar to the unimpaired group. HE is manifested by a continuum of symptoms and the categorisation into grades is based on detectable symptoms and not on prognosis, recognising full well that sometimes the diagnosis of Grade 1 HE may be difficult to make with certainty. However, the prognostic differences between the groups with grade 1 HE and mHE described may provide the argument to try and separate the ‘Covert’ group into its subcomponents, even if such a distinction is difficult.

The diagnosis of mHE is based on both neurophysiological and psychometric tests. We classified patients as having mHE based on at least one abnormal test using three different currently validated testing strategies. It is not clear which cirrhotic patients will display abnormalities in the different tests and the EASL/AASLD guidelines suggest that when testing for mHE at least two validated testing strategies should be utilized, one of them being PHES [8, 22]. Pathophysiologically, it is also known that many brain regions are differently affected in individual patients supporting the notion that mHE may manifest with multidimensional neuropsychiatric symptoms ranging from abnormalities in cognition, attention, memory, anxiety and depression. Thus, it was not surprising that about half of the mHE patients had only one abnormal test to allow diagnosis of mHE. Only about 20% patients had abnormal outcomes in all three tests. In an additional analysis, we re-classified our patients to diagnose mHE as defined by abnormality of two tests (as suggested by the EASL/AASLD guidelines), one of them being PHES. The overall results were similar in terms of complications and mortality between the three groups (Table 5).

**Table 5:** Baseline characteristics of patients with cirrhosis according to the hepatic encephalopathy classification (2 tests abnormal for diagnosis of mHE).

	No HE	mHE*	Grade 1 HE	P-value
	n = 45	n = 17	n = 44	(all 3 groups)
Sex: m/f (%)	62/38	62/38	59/41	P = 0.44
Age (year)	59 ± 7	58 ± 12	58 ± 12	P = 0.83
MELD	14 ± 5	14 ± 7	16 ± 6	P = 0.17
Ascites: 0/1/2 (%)	16/20/64	23/6/71	0/2/98	P = 0.001
Bilirubin (μmol/L)	70 ± 74	111 ± 122	65 ± 49	P = 0.09
INR	1.5 ± 0.3	1.5 ± 0.4	1.5 ± 0.3	P = 0.90
Albumin (g/dL)	2.8 ± 0.6	2.9 ± 0.7	2.9 ± 0.5	P = 0.63
Ammonia (μmol/L)	56 ± 14	56 ± 15	62 ± 12	P = 0.06
Sodium (mmol/L)	136 ± 6	136 ± 4	135 ± 5	P = 0.59
Creatinine (μmol/L)	81 ± 47	68 ± 51	81 ± 43	P = 0.60
WBC count (x 10^9^/L)	5.5 ± 2.9	7.0 ± 2.5	7.4 ± 4.8	P = 0.06
Bacterial DNA n (%)	14 (31)	5 (29)	25 (57)	P = 0.03
Neut. resp. burst (%)	15 ± 14	9 ± 9	22 ± 22	P = 0.01
Phagocytosis (GMFI)	83 ± 14	82 ± 14	78 ± 10	P = 0.25
Complications n (%)	12 (27)	8 (47)	28 (64)	P = 0.02
Death n (%)	1 (2)	2 (12)	9 (20)	P = 0.03

*at least 2 abnormal tests (one being PHES) for diagnosis of mHE.

HE = hepatic encephalopathy, MELD = Model for End-Stage Liver Disease, WBC = white blood cell count, GMFI = geometric mean of fluorescence intensity.

Of the patients with at least 2 abnormal tests, almost 70% had grade 1 HE. Also WBC levels were higher in these patients demonstrating the important synergetic effect of inflammation in the development of HE. Complication rate and mortality were similar when comparing patients with abnormal EEG and PHES only, while having at least two abnormal tests, increased the risk significantly. Similar findings were recently reported by Montagnese et al and Lauridsen et al [23, 24]. Any division of HE into grades is clearly a simplification based on symptoms and not on prognosis. A possible solution to better discriminate patients who do not have ‘overt’ HE may be to classify them according to number of abnormal tests. In this model, a patient with ‘Covert HE’ may be classified into a low-risk group, identified by the presence of one abnormal test, whereas the presence of 2 abnormal tests would identify a high-risk group instead of the challenging diagnosis of Grade 1 HE. Hereby, it might be possible to distinguish between groups with different prognosis. This hypothesis will need to be tested in future studies.

Although the literature suggests that the occurrence of overt HE is more common in mHE compared to unimpaired patients [5, 6], the present study did not show a significant difference. This discrepancy may be explained by either too few events in our study due to short period of observation or by patients with previous history of ‘overt HE’ being included in the other studies. In fact, 28% of patients with mHE had previous episodes of ‘overt HE’ compared with 4% of the unimpaired patients in the study by Hartmann et al [6]. Alternatively, it is possible that at least some of the patients diagnosed with mHE in those previous studies may have had undiagnosed Grade 1 HE despite the fact that ‘presence of HE’ was an exclusion criteria. As suggested by the EASL/AASLD HE guidelines, that unless specific examination is performed to look for evidence of Grade 1 HE, its diagnosis can be missed [7].

The data also showed that infection was the most common complication in patients with Grade 1 HE. Conversely, MELD scores and synthetic liver function tests including ammonia, albumin, INR and bilirubin levels were similar in the three groups. The underlying mechanism linking the presence of Grade 1 HE and increased complications and mortality, despite otherwise similar severity of liver disease, is uncertain. Although white blood cell counts were not different between patients with mHE and Grade 1 HE, patients with Grade 1 HE had more frequent evidence of bacterial translocation and higher spontaneous neutrophil respiratory burst compared to patients with mHE. Neutrophil dysfunction was also an independent risk factor of mortality. These findings indicate a potential mechanistic link between neutrophil dysfunction and increased risk of infection. Studies in animal models and in patients with stroke suggest that immune dysfunction may well be a consequence of brain dysfunction [25–28]. In animal models, mild occlusion of the middle cerebral artery was shown to result in immune dysfunction resulting in reduced ability to clear infection that was introduced iatrogenically into the lungs [25]. In keeping with this observation, there is increased risk of bacterial infection in patients with stroke, which is also one of the main causes of death of patients with stroke. Therefore, it is possible that HE may independently result in neutrophil dysfunction making patients more susceptible to infection rather than being its consequence. This hypothesis will need to be confirmed in future studies.

Increased spontaneous neutrophil burst was predictive of subsequent infection and organ dysfunction in patients with alcoholic hepatitis [10]. Bacterial DNA has been demonstrated to result in neutrophil activation [29] and studies suggest that endotoxemia is one of the possible causes of spontaneous neutrophil burst and hypothesize that the underlying mechanism may be increased gut permeability, which is a well-known feature of cirrhosis [10, 30, 31]. Increased bacterial translocation is common in patients with ascites and its frequency correlates with the severity of liver failure [32, 33]. The Grade 1 HE patients had higher prevalence of ascites and also a non-significantly higher rate of bacterial DNA in their plasma [19]. The mechanism of the higher frequency of ascites and bacterial DNA in Grade 1 HE is not clear but it is interesting to consider the potential role of the autonomic nervous system in modulating the severity of gut inflammation [34]. Newton et al. suggested that in patients with primary biliary cirrhosis with cognitive impairment, autonomic dysfunction is prevalent [35]. Therefore, the observation of Wiest et al. that splanchnic sympathectomy in an animal model reduced bacterial translocation of gram negative organisms suggests a possible link between bacterial translocation and HE [36]. This hypothesis will need to be tested in suitable models.

We observed no difference in ammonia levels between patients with mHE and Grade 1 HE. Ammonia levels were not an independent predictor of outcome, although values were higher in the patients who developed complications requiring hospital admission and those who died. Ammonia has been shown to be important in the pathogenesis of neutrophil dysfunction [11] but we did not observe any direct correlation between the severity of neutrophil dysfunction and ammonia levels indicating other operative mechanisms underlying its pathogenesis. In a recent study, Montagnese et al showed that EEG abnormalities in patients with cirrhosis were associated with higher ammonia levels compared to patients with impaired PHES performance, pointing to possibly different mechanisms underlying the mHE phenotypes [37]. This finding was confirmed in our study as patients with abnormal EEG also had higher ammonia levels compared to patients with abnormal PHES test. These data provide further evidence that from the pathophysiological perspective, mHE is also a heterogenous entity and therefore the treatment approaches for patients presenting with different abnormalities in psychometric tests may well be different.

In conclusion, the results of this study show for the first time that ‘Covert HE’ is a heterogeneous entity comprising two clinically and pathophysiologically distinct syndromes that should be described separately until more data are available. Infection is the most common complication necessitating hospital admission in Grade 1 HE patients and this is associated with higher rates of bacterial translocation and neutrophil dysfunction. Further studies should address whether this is a cause or consequence of HE.

## References

[1] Cordoba J, Ventura-Cots M, Simon-Talero M, Amoros A, Pavesi M, Vilstrup H, et al Characteristics, risk factors, and mortality of cirrhotic patients hospitalized for hepatic encephalopathy with and without acute-on-chronic liver failure (ACLF). J Hepatol. 2014;60(2):275–81. 10.1016/j.jhep.2013.10.004 . 24128414

[2] Bustamante J, Rimola A, Ventura PJ, Navasa M, Cirera I, Reggiardo V, et al Prognostic significance of hepatic encephalopathy in patients with cirrhosis. J Hepatol. 1999;30(5):890–5. . 10365817 10.1016/s0168-8278(99)80144-5

[3] Jepsen P, Ott P, Andersen PK, Sorensen HT, Vilstrup H. Clinical course of alcoholic liver cirrhosis: a Danish population-based cohort study. Hepatology. 2010;51(5):1675–82. 10.1002/hep.23500 . 20186844

[4] Stewart CA, Malinchoc M, Kim WR, Kamath PS. Hepatic encephalopathy as a predictor of survival in patients with end-stage liver disease. Liver Transpl. 2007;13(10):1366–71. 10.1002/lt.21129 . 17520742

[5] Romero-Gomez M, Boza F, Garcia-Valdecasas MS, Garcia E, Aguilar-Reina J. Subclinical hepatic encephalopathy predicts the development of overt hepatic encephalopathy. Am J Gastroenterol. 2001;96(9):2718–23. 10.1111/j.1572-0241.2001.04130.x . 11569701

[6] Hartmann IJ, Groeneweg M, Quero JC, Beijeman SJ, de Man RA, Hop WC, et al The prognostic significance of subclinical hepatic encephalopathy. Am J Gastroenterol. 2000;95(8):2029–34. 10.1111/j.1572-0241.2000.02265.x . 10950053

[7] Ortiz M, Jacas C, Cordoba J. Minimal hepatic encephalopathy: diagnosis, clinical significance and recommendations. J Hepatol. 2005;42 Suppl(1):S45–53. 10.1016/j.jhep.2004.11.028 . 15777572

[8] American Association for the Study of Liver D, European Association for the Study of the L. Hepatic encephalopathy in chronic liver disease: 2014 practice guideline by the European Association for the Study of the Liver and the American Association for the Study of Liver Diseases. J Hepatol. 2014;61(3):642–59. 10.1016/j.jhep.2014.05.042 . 25015420

[9] Bajaj JS, Cordoba J, Mullen KD, Amodio P, Shawcross DL, Butterworth RF, et al Review article: the design of clinical trials in hepatic encephalopathy—an International Society for Hepatic Encephalopathy and Nitrogen Metabolism (ISHEN) consensus statement. Aliment Pharmacol Ther. 2011;33(7):739–47. 10.1111/j.1365-2036.2011.04590.x 21306407 PMC3971432

[10] Mookerjee RP, Stadlbauer V, Lidder S, Wright GA, Hodges SJ, Davies NA, et al Neutrophil dysfunction in alcoholic hepatitis superimposed on cirrhosis is reversible and predicts the outcome. Hepatology. 2007;46(3):831–40. 10.1002/hep.21737 . 17680644

[11] Shawcross DL, Wright GA, Stadlbauer V, Hodges SJ, Davies NA, Wheeler-Jones C, et al Ammonia impairs neutrophil phagocytic function in liver disease. Hepatology. 2008;48(4):1202–12. 10.1002/hep.22474 . 18697192

[12] Ong JP, Aggarwal A, Krieger D, Easley KA, Karafa MT, Van Lente F, et al Correlation between ammonia levels and the severity of hepatic encephalopathy. Am J Med. 2003;114(3):188–93. . 12637132 10.1016/s0002-9343(02)01477-8

[13] Tranah TH, Vijay GK, Ryan JM, Shawcross DL. Systemic inflammation and ammonia in hepatic encephalopathy. Metab Brain Dis. 2013;28(1):1–5. 10.1007/s11011-012-9370-2 . 23224356

[14] Shawcross DL, Sharifi Y, Canavan JB, Yeoman AD, Abeles RD, Taylor NJ, et al Infection and systemic inflammation, not ammonia, are associated with Grade 3/4 hepatic encephalopathy, but not mortality in cirrhosis. J Hepatol. 2011;54(4):640–9. 10.1016/j.jhep.2010.07.045 . 21163546

[15] Tritto G, Bechlis Z, Stadlbauer V, Davies N, Frances R, Shah N, et al Evidence of neutrophil functional defect despite inflammation in stable cirrhosis. J Hepatol. 2011;55(3):574–81. 10.1016/j.jhep.2010.11.034 . 21236309

[16] Van der Rijt CC, Schalm SW, De Groot GH, De Vlieger M. Objective measurement of hepatic encephalopathy by means of automated EEG analysis. Electroencephalogr Clin Neurophysiol. 1984;57(5):423–6. . 6201336 10.1016/0013-4694(84)90071-3

[17] Weissenborn K, Ennen JC, Schomerus H, Ruckert N, Hecker H. Neuropsychological characterization of hepatic encephalopathy. J Hepatol. 2001;34(5):768–73. . 11434627 10.1016/s0168-8278(01)00026-5

[18] Kircheis G, Wettstein M, Timmermann L, Schnitzler A, Haussinger D. Critical flicker frequency for quantification of low-grade hepatic encephalopathy. Hepatology. 2002;35(2):357–66. 10.1053/jhep.2002.30957 . 11826409

[19] Such J, Frances R, Munoz C, Zapater P, Casellas JA, Cifuentes A, et al Detection and identification of bacterial DNA in patients with cirrhosis and culture-negative, nonneutrocytic ascites. Hepatology. 2002;36(1):135–41. 10.1053/jhep.2002.33715 . 12085357

[20] Kamath PS, Wiesner RH, Malinchoc M, Kremers W, Therneau TM, Kosberg CL, et al A model to predict survival in patients with end-stage liver disease. Hepatology. 2001;33(2):464–70. 11172350 10.1053/jhep.2001.22172

[21] Pugh RN, Murray-Lyon IM, Dawson JL, Pietroni MC, Williams R. Transection of the oesophagus for bleeding oesophageal varices. Br J Surg. 1973;60(8):646–9. . 4541913 10.1002/bjs.1800600817

[22] Vilstrup H, Amodio P, Bajaj J, Cordoba J, Ferenci P, Mullen KD, et al Hepatic encephalopathy in chronic liver disease: 2014 Practice Guideline by the American Association for the Study of Liver Diseases and the European Association for the Study of the Liver. Hepatology. 2014;60(2):715–35. 10.1002/hep.27210 . 25042402

[23] Montagnese S, Balistreri E, Schiff S, De Rui M, Angeli P, Zanus G, et al Covert hepatic encephalopathy: agreement and predictive validity of different indices. World J Gastroenterol. 2014;20(42):15756–62. 10.3748/wjg.v20.i42.15756 25400460 PMC4229541

[24] Lauridsen MM, Schaffalitzky de Muckadell OB, Vilstrup H. Minimal hepatic encephalopathy characterized by parallel use of the continuous reaction time and portosystemic encephalopathy tests. Metab Brain Dis. 2015 10.1007/s11011-015-9688-7 . 26016624

[25] Meisel C, Schwab JM, Prass K, Meisel A, Dirnagl U. Central nervous system injury-induced immune deficiency syndrome. Nat Rev Neurosci. 2005;6(10):775–86. 10.1038/nrn1765 . 16163382

[26] Dirnagl U, Klehmet J, Braun JS, Harms H, Meisel C, Ziemssen T, et al Stroke-induced immunodepression: experimental evidence and clinical relevance. Stroke. 2007;38(2 Suppl):770–3. 10.1161/01.STR.0000251441.89665.bc . 17261736

[27] Emsley HC, Hopkins SJ. Post-stroke immunodepression and infection: an emerging concept. Infect Disord Drug Targets. 2010;10(2):91–7. . 20166972 10.2174/187152610790963528

[28] Prass K, Meisel C, Hoflich C, Braun J, Halle E, Wolf T, et al Stroke-induced immunodeficiency promotes spontaneous bacterial infections and is mediated by sympathetic activation reversal by poststroke T helper cell type 1-like immunostimulation. J Exp Med. 2003;198(5):725–36. 10.1084/jem.20021098 12939340 PMC2194193

[29] El Kebir D, Jozsef L, Filep JG. Neutrophil recognition of bacterial DNA and Toll-like receptor 9-dependent and -independent regulation of neutrophil function. Arch Immunol Ther Exp (Warsz). 2008;56(1):41–53. 10.1007/s00005-008-0008-3 . 18250968

[30] Etheredge EE, Spitzer JA. Chronic endotoxemia reversibly alters respiratory burst activity of circulating neutrophils. J Surg Res. 1993;55(3):261–8. 10.1006/jsre.1993.1138 . 8412108

[31] Bohmer RH, Trinkle LS, Staneck JL. Dose effects of LPS on neutrophils in a whole blood flow cytometric assay of phagocytosis and oxidative burst. Cytometry. 1992;13(5):525–31. 10.1002/cyto.990130512 . 1321708

[32] Albillos A, de la Hera A, Gonzalez M, Moya JL, Calleja JL, Monserrat J, et al Increased lipopolysaccharide binding protein in cirrhotic patients with marked immune and hemodynamic derangement. Hepatology. 2003;37(1):208–17. 10.1053/jhep.2003.50038 . 12500206

[33] Cirera I, Bauer TM, Navasa M, Vila J, Grande L, Taura P, et al Bacterial translocation of enteric organisms in patients with cirrhosis. J Hepatol. 2001;34(1):32–7. . 11211904 10.1016/s0168-8278(00)00013-1

[34] Straub RH, Wiest R, Strauch UG, Harle P, Scholmerich J. The role of the sympathetic nervous system in intestinal inflammation. Gut. 2006;55(11):1640–9. 10.1136/gut.2006.091322 17047110 PMC1860105

[35] Newton JL, Hollingsworth KG, Taylor R, El-Sharkawy AM, Khan ZU, Pearce R, et al Cognitive impairment in primary biliary cirrhosis: symptom impact and potential etiology. Hepatology. 2008;48(2):541–9. 10.1002/hep.22371 . 18563843

[36] Worlicek M, Knebel K, Linde HJ, Moleda L, Scholmerich J, Straub RH, et al Splanchnic sympathectomy prevents translocation and spreading of E coli but not S aureus in liver cirrhosis. Gut. 2010;59(8):1127–34. 10.1136/gut.2009.185413 . 20519743

[37] Montagnese S, Biancardi A, Schiff S, Carraro P, Carla V, Mannaioni G, et al Different biochemical correlates for different neuropsychiatric abnormalities in patients with cirrhosis. Hepatology. 2011;53(2):558–66. 10.1002/hep.24043 . 21274876

